# Bearing witness through pandemic borders and film: convergent media, mobility and *Windrush Betrayal*

**DOI:** 10.1007/s10708-022-10652-1

**Published:** 2022-05-09

**Authors:** Susan P. Mains

**Affiliations:** Geography and Environmental Science, School of Social Sciences, University of Dundee, Dundee, DD1 4HN UK

**Keywords:** Convergent media, Caribbean Diaspora, Film, Windrush, Immigration

## Abstract

This paper examines the role of convergent media and the film, *Windrush Betrayal* (2020), in representing and challenging borders, mobilities and UK government immigration policies. Print and digital news provide important contexts for exploring media geographies of current events. Documentary film is also a provocative medium for investigative analyses that go beyond more general headline reporting. This study seeks to expand on earlier studies by examining how complementary mediums such as digital news media and film can respond to each other and become part of dynamic transnational conversations around place and identity. Media formats have been pushed to incorporate new settings and styles as Covid-19 restrictions have been implemented and alternative approaches utilised in media production. Adopting innovative techniques for filming in response to pandemic restrictions, *Windrush Betrayal* illustrates the ongoing impacts of immigration policies on Caribbean Diaspora populations in the UK. This paper provides a timely opportunity to tease out the ways in which changes in government immigration policies, media work practices and the production of migration narratives can highlight hidden geographical stories and marginalised voices.

Print and digital news provide important contexts for exploring media geographies of current events. In particular, documentary film is a provocative medium for investigative analyses that go beyond more general headline reporting. An analysis of the ways in which complementary mediums such as digital news media and film respond to each other and become part of dynamic conversations, provides timely illustrations of how we represent immigration, race, place and identity. Media formats have been pushed to incorporate new settings and styles as Covid-19 restrictions have been implemented and alternative approaches utilised in media production. This paper examines the role of convergent media, government policies, and the film, *Windrush Betrayal* (2020), in representing and challenging borders, mobilities, and UK government immigration policies. Examining convergent media provides a timely opportunity to tease out the ways in which changes in government policies, media work practices and the production of migration narratives can highlight hidden geographical stories and marginalised voices.

The analysis below highlights how representational and material borders, in combination with uneven mobilities, are key components of media narratives around immigration monitoring and the UK’s deportation strategies. Two key questions are central:

In what ways do convergent media facilitate a shift in our understanding of “government” and “news” spaces?

How do film representations, particularly *Windrush Betrayal*, combine with news coverage to create counter spaces and narratives around immigration and the Caribbean Diaspora in the UK?

In order to address these questions, the paper is divided into four sections. The first section explores media convergence during the Covid-19 pandemic and borders between public and private spaces. This is then followed by a discussion of the Home Office’s “Hostile Environment” procedures and the contradictory mobilities represented prior to *Windrush Betrayal*. Adopting new techniques for filming in response to pandemic restrictions, *Windrush Betrayal* illustrates the ongoing negative impacts of immigration policies on Caribbean Diaspora populations in the UK. The third section examines the connections between newspaper investigations, the *Windrush Lessons Learned Review* (Williams, 2020) and media narratives in *Windrush Betrayal*. The concluding section provides a brief reflection on what the examples discussed may offer in terms of inclusive and pro-active convergent media news practices, and the role of film geographies.

## Pandemic reporting: media convergence and blurring borders

Media convergence refers to processes through which different media formats become intertwined and, ideally, streamline the processes of sharing content. In the context of news coverage, for example, we have seen increasing connections being made between print editions, website-based news sites and social media coverage. A discussion about a specific event on social media (for example a controversial announcement on Twitter or a story on Instagram), may then be picked up and discussed on terrestrial television news and later covered in print press the following day (or in an evening edition). Media content has also been less tied to a specific kind of technology (for example, online news coverage can be viewed on smart televisions, tablets, or mobile telephones), which, in turn has altered the geography of producing and viewing news. Audiences can view more in-depth news documentaries at a time of their choosing, are not tied to one location to view materials and may fact check as they are doing so by accessing other websites and related social media discussions. As Cupples and Glynn (2016, 60) note in their discussion of convergent media and indigenous identities in New Zealand, these formats open up the opportunity to decentralise, diversify and challenge exclusionary representations:

Key features of the twenty-first-century media environment include the digital proliferation of distribution channels associated with post-broadcast media forms, the expansion of new participatory media cultures, and intensified geographical connectedness and possibilities for transcultural dialogue as images and discourses spill interactively across a multiplicity of networked screens and platforms.

In conjunction with the movement and interaction of news content across a range of formats, there has also been increasing efforts to standardise the style and tone of coverage within media organisations. News outlets have a recognisable brand—corporate logos, reporting styles and key spokespeople are easily recognisable—and with this familiarity comes an expectation of the kinds of stories and perspectives that will be communicated. By making content more uniform and recognisable there is, therefore, a centralising tendency that runs in tandem with the decentralisation of the formats and devices through which we access this coverage. While there has been a growing body of research examining the challenges posed for diverse views through this media centralisation (Barnett et al., 2017), the possibilities of tensions between media formats also offer the opportunities of using familiar news brands in resistant ways.

The Covid-19 pandemic has illustrated several ways in which relatively mainstream media outlets have been required to stretch and reconfigure how programmes, films, and live news are created and shared. Social distancing policies have resulted in limitations to who can travel and how many people may meet up at specific locations, resulting in restrictions on producing media and viewing media in public venues. These restrictions have varied in terms of location regionally and globally, but the effects have nonetheless been widespread.

One significant change has been a shift from largely studio-based news reporting to the incorporation of live reports via software such as Zoom and Microsoft Teams with topical “experts” based at their own homes. This more “ad-hoc” content has facilitated the incorporation—and at times active embracing—of more unpredictable and less on-brand forms of coverage. In a relatively short timeframe (of approximately a few months), reports from peoples’ kitchens, living rooms or temporary home-offices became a ubiquitous component of media coverage in various media genres. What was previously viewed as a somewhat limited, and at times quirky form of reporting, has now become part of the everyday. With this technical shift, (curated) domestic spaces have become widely visible. Home web cameras or laptops have been placed in locations that include backgrounds largely pointing at bookshelves, artwork, or blank walls (although there are occasional exceptions that receive extensive interrogation in related social media threads, particularly focusing on bookshelves! (Hess, 2020)). In relation to news coverage, this has resulted in the extension of the newsroom to these somewhat cordoned off spaces of private homes. The home is frequently depicted as being a space of emotion and privacy, however, the increasing visibility of home reporting is an interesting geographical shift that could also reconfigure images of domestic spaces as incorporating pockets of ‘neutrality’ for the purposes of broadcasting in socially distanced times.

Attempts to “loosen” the formats and content of mainstream media has emerged in tandem with narrowly selected spokespeople. The reduced need to physically be in particular locations in order to participate in live news coverage or investigative documentary film reports, also poses possibilities for diversifying the range of experts who are represented in media coverage. For many years there has been ongoing critiques of the lack of racial, gendered and regional diversity in mainstream British news media (see, for example, Hall, 1997; Mains, 2003; Holloway, 2015; hooks, 2015) and the shift to more mobile forms of reporting can provide an important avenue for addressing more socially inclusive content. The increasingly high quality and widely available materials produced through social media and by “citizen reporters” in recent years has laid a challenge at the feet of mainstream directors and programmers.

Critical approaches towards media images can be considered part of a process of decolonising popular representations. The process of decolonisation involves interrogating unequal power relationships, widening the possibilities for who produces media, diversifying expectations in terms of audiences, and providing alternative narratives of place and identity that document and engage with a wide range of experiences. Within the context of mainstream media, discussions around the process of decolonisation have intensified since the emergence of the Covid-19 pandemic in tandem with discussions around the Black Lives Matter (BLM) movement—a movement challenging racial injustice, discrimination and inequalities—and growing protests around the tacit approval of built landscapes that fail to reflect on the problematic, and racist geographies and histories they embody. One of the most obvious examples of this in the UK recently has been media discussions addressing protests challenging public statues dedicated to historical figures who were actively engaged with colonialist activities, such as slavery (Alexander, 2020). Media coverage of protests challenging racial injustice highlight the partial and uneven approaches utilised when representing new social movements. As Umamaheswar (2020) explains in her recent analysis examining media coverage of BLM: “despite representing the goals of the movement faithfully through the perspectives of protesters, the movement’s goal of raising awareness of racial injustice was side-lined in favour of more sensational stories linking the movement with violence against police.”

While there are some media stories that go into greater depth about complex issues racial injustice protestors are highlighting, Umamaheswar (2020) illustrates that a “protest paradigm” utilised in media coverage tends to situate these stories within a narrative that disproportionately concentrates on crime and rioting, thus depicting the movements as a potentially threatening force. The use of key phrases, placement of cameras and framing of newsprint photographs from a specific perspective utilises convergent media to reinforce a combative narrative, and one in which the viewer or reader is encouraged to align themselves with (so-called) experts who often have limited knowledge of the day-today challenges faced by those pushing for social and political change. Establishment media and governments utilise partial narratives to dehumanise and criminalise protestors in the eyes of the wider public. This is not however, an inevitability, as the analysis in the following section highlights, convergent media coverage of race and protest also offers counter-narratives that challenge the generalising representations of media coverage and question the borders between policy and everyday life.

Protest takes various spatial and temporal forms. Although protests are commonly featured through media coverage of street marches, demonstrators outside of buildings and/or through trending social media on specific dates, these are foci on relatively limited public spaces and daily timeframes. If we shift our focus to the act of producing and viewing media, we can also see that these practices in themselves can be acts of resistance challenging and reframing popular narratives. The timespan of media—for example, documentary film—can then be extended through individual viewings and shared screenings that connect with and extend key themes raised by brief news stories or more superficial content. In addition, the settings in which documentary films are set may expand the mediated geographies of protest: marginalised residents or artists may be interviewed at home, in a studio, at school, or in a wide range of locations that are not stereotypically used as shorthand for “a protest site.” The spatial and temporal borders and mobilities of protest representations shift, and so too do the spaces in which we can engage with experts.

In addition to producing media content that diversifies images of experts and turns home spaces into satellite newsrooms (to some extent), pandemic reporting has highlighted the complex nature of news. Recent pandemic representations of current events have highlighted the contradictory nature of how we envision news and domestic space. While images of home are being expanded to include public facing centres of timely information (in the form of kitchens, living rooms, attic rooms, etc.), there are frequently moments where this professionalisation of the home is disrupted. In pandemic times, children run in and disrupt an interview, people express upset in their living room, interviewees in different locations speak over each other, and technology breaks down disrupting reporters’ broadcasts, all resulting in impromptu off-scripted conversations. These are moments of slippage that highlight the interconnections of emotional and professional spaces. The dramatic circumstances of the Covid-19 pandemic have highlighted the power of emotion and its relevance to news reporting has become increasingly evident (although mobilised for a variety of purposes). Individual responses to recent challenging circumstances, including constrained opportunities for travel, experiences of social distancing and impacts of injustice (through, for example, video diaries or staged zoom plays (see, for example, UNICEF, 2020; BBC Travel Show, 2020; Bush Theatre, 2020), have become a key point of sharing, and providing connections with audiences in new ways. These connections provide a documentation, sharing and analysis of emotions as a central component of changing responses to pandemic conditions.

Emotion has always been a component of media, and recent news content draws on intimate and affective media geographies in ways that blur work and home geographically, attempting to provide managed presenters at-a-distance, while being flexible enough to include more personal experiences. While it could be argued that individual experiences are being utilised as a largely convenient ratings strategy in the example of *The Protest Series* (Bush Theatre, 2020) and *Windrush Betrayal* (2020) discussed below, these representations are deployed in disruptive, progressive and subversive ways. The ability to bring actors’ expressions into greater focus or the shift to broadcasters filming at home during lockdown—making the private public—is echoed in *Windrush Betrayal*. The latter is also particularly striking because these domestic spaces that are often symbolic of security and familiarity are highlighted as potentially vulnerable to discriminatory state practices.

## Representing hostile environments: media, movements and shifting borders

In 2017 it emerged that people who had moved from the Caribbean to the UK—often decades before as young children travelling with their parents—were being charged by the British government for living in the country without the appropriate legal status. In many cases these were British residents and citizens who were legally residing in the country, but had become victims of a punitive immigration ministry, the Home Office. This ministry was actively promoting an increasingly problematic approach towards immigration that emerged out of a government strategy spearheaded in 2012 by the then Home Secretary, Theresa May. The policy was heralded by the Conservative-led government as actively creating a “Hostile Environment.” A key component of this environment involved ensuring access to key government services—state education, healthcare, employment, and financial resources—was impossible for people who were unable to provide confirmation of migration status. In effect, this policy relied on incorporating a range of state agencies as indirect border and immigration agents, and as a consequence since this period, has led to the increasingly precarious existence for those without extensive residency documentation (and even for some who do have proof of UK residence over several decades). The stretching of physical borders to incorporate additional institutions, representations and social spaces has been used by other national governments (and frequently met with significant opposition). Migrant advocates and geographers have noted that such an approach stretches borders in ways that increasingly impinges on the everyday lives of residents and produces unequal mobilities and insecurities (Mains, 2018).

While unhelpful (or aggressive) professionals and intimidating buildings act as extended symbols of border control, recent convergent media representations are also a critical component of reinforcing and challenging boundary making practices. Sensationalist television news stories speaking of potential migrant “threats,” feature films with migrant figures who are represented through stereotypical negative characterisations, or inflammatory language used in policy conversations, are combined with the physical deployment of hostile media through posters and warning signs to create fear and discomfort. “Go home” vans were deployed in London boroughs in 2013, with large adverts on their sides asking: “In the UK illegally? Go home or face arrest” (Savage, 2018). These vans were assigned to racially diverse areas and viewed by many as an antagonistic gesture—a discursive and physical incursion into the built and social city landscape. They were also symbols of contradictory and contested mobilities: they represented the increasingly mobile reach of the Home Office while attempting to limit the mobility of specific migrant and resident populations. Activities such as those above have relied on borders being policed and mapped onto specific spaces and migrant identities, and as we see in the discussion below, one such group in the UK is the “Windrush Generation.”

**Fig. 1:**
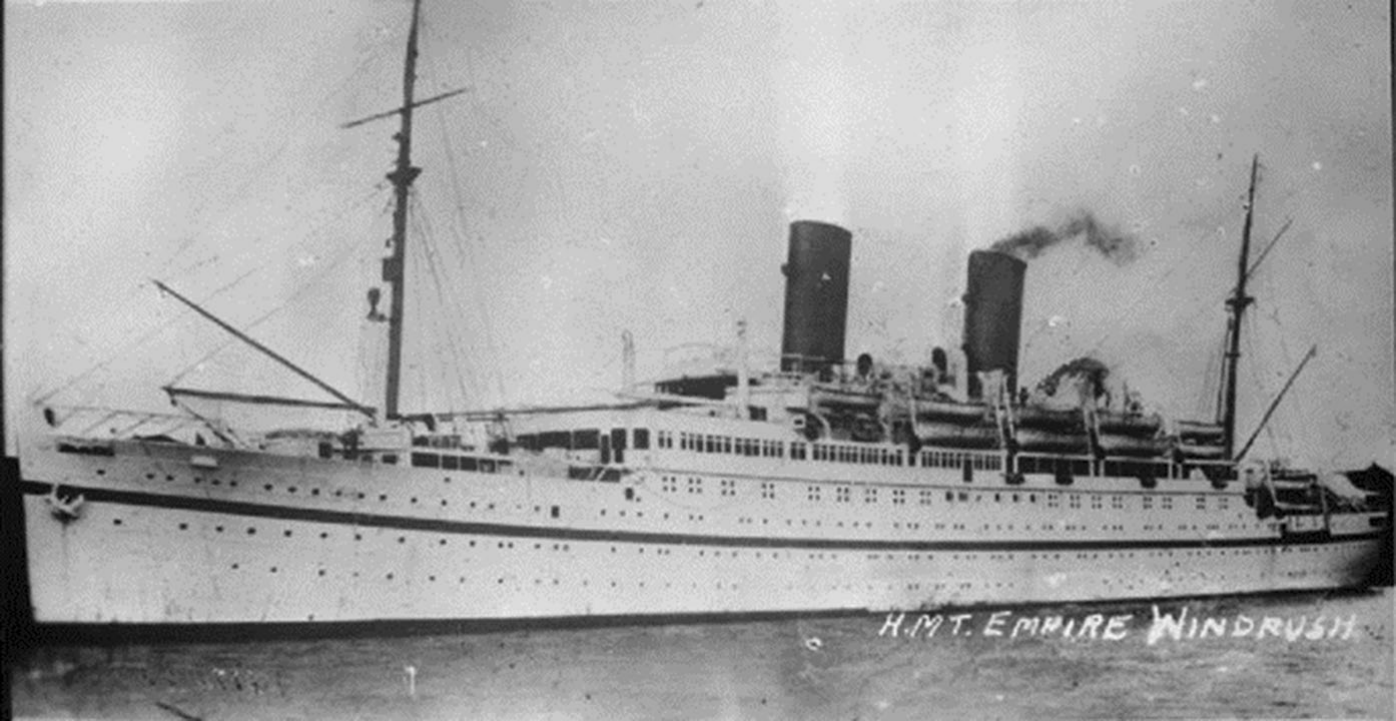
*HMT Empire Windrush,* 1945–54 (FL9448, Imperial War Museum/Creative Commons)

The Windrush Generation refers to those who migrated from the Caribbean to the UK as part of the post-war re-building strategy, specifically during the period 1948–1971. The *HMT Empire Windrush* was the ship that sailed from Kingston, Jamaica and landed at the Port of Tilbury, London in 1948 with 1027 passengers (and two stowaways) on board, and just over 800 people giving a Caribbean country as their residence prior to sailing. Passengers included former servicemen who had fought as part of the allied forces and were planning to re-join on arrival in Britain, young women trained as teachers and nurses, and additional travellers who had been encouraged to take up spaces available through advertisements placed in the Jamaican press prior to departure (Fig. 1).

Black and white film images of *HMT Empire Windrush* have become iconic: a shorthand for mobility and opportunity, separation from family, geopolitics and commonwealth relations, travelling to the familiar “Mother Country” and an unknown future. In the 1940s these images appeared in a context where there was limited cross-over in media from one medium to another—i.e., in terms of being able to view moving images in different contexts—and this meant that the popular Pathé News films of the time were central to encouraging audiences’ spatial imaginaries of migration, travel and government responsibility. The upbeat tone of the initial arrival films and the call to “welcome” new arrivals are often referred to as signalling an important contribution to the cultural diversity of the UK. Reporters conduct brief interviews with passengers, learning of their hopes for work. In one film the interviewer encourages a passenger—the already successful musician, Lord Kitchener—to sing a calypso introduction to their new home, combining the sounds from both sides of the Atlantic (British Pathé, 1948).

At the time of their release, Pathé News films provided a vivid image of Caribbean migrants to the UK. Shown as weekly newsreels to cinema audiences, Pathé’s short films provided a popular (approximately 10 min) overview of current events, with a post-war emphasis being placed on those travelling from Jamaica, which was still a part of Britain’s colonial empire (until official Independence in 1962). The ship’s arrival was not only an abstract vessel or simply a mode of transportation, but a context through which individual travellers spoke about their hopes for their futures. The footage also reinforced the notion that Jamaica and the UK were separated/connected by permeable borders and the British government had a responsibility to ensure the welcome and safety of Caribbean-based British citizens—particularly those who arrived on its shores. Reviewing these films several decades later highlights the poignancy and overwhelming injustices that many migrants have faced in the intervening years—housing discrimination, police violence, limited employment opportunities and disproportionate incarceration—and the dramatic shift in the symbolism of the *HMT Empire Windrush* as an emblem of hope, connection and opportunity to one of dislocation, exclusion and discrimination.

Film plays an important role in challenging anti-immigrant sentiment and increasingly restrictive government policies towards the Caribbean since the post-World War II period. While official news reel films, such as those shown by British Pathé, adopt a largely upbeat and sanitised coverage of migrants’ experiences, documentaries and feature films provide a parallel and challenging narrative, elaborating on the emotional cartographies of Black British life, and the everyday ongoing discrimination that imbues living in the UK. Clark (2014) notes in an overview of a series of wide-ranging films exploring Black British experiences during the last sixty years:

Following a number of issue-based films that addressed the black British experience in the 1950s and 60s (*Pool of London, Sapphire, Flame in the Streets*), the first wave of films to genuinely focus on a new generation of post-war, Windrush-era arrivals and their children in Britain began in 1975 with the release of Horace Ové’s brilliant *Pressure*.

The 1980s saw a surge in activity for black British production and representation, encouraged in part by the arrival of Channel 4 (which put diverse programming at the centre of its remit) in 1982. Since then, due to a combination of factors (lack of training and funding, exodus of talent to the United States), there’s been a cycle of frustration in maintaining and developing the black British presence on film.

Despite these challenges, since 2015 there has been a growing body of Black British film, traversing cinema, television dramas and documentaries, and digital media on a variety of platforms. Broadcast in 2021 by the *BBC* on terrestrial television and on its online iPlayer, Steve McQueen’s *Small Axe* series of hour- long films, provide an important historical geography to the crisis illustrated by the *Windrush Betrayal* discussed below. Described as “Love letters to black resilience and triumph in London's West Indian community…Vivid stories of hard-won victories in the face of racism” (BBC iPlayer, 2021), this series of films also intersects with *Sitting in Limbo* (2020)and recent digital films being produced as part of BLM protests in 2020–2021 and calls for more inclusive mainstream media in tandem with the promotion of independent and experimental productions.

## Resistant film geographies: *The Lessons Learned Review*, *The Guardian, and Windrush Betrayal*

Films run in parallel to other forms of media to produce and challenge narratives of place, mobility and identity. Although frequently produced in distinct settings, through specific technologies and distribution networks, film increasingly responds to and challenges news stories in other formats. This section analyses the ways in which the film *Windrush Betrayal* intersects with related policy, film and news media coverage of Caribbean communities in the UK, and in doing so creates mediated geographies and counter-narratives of space and immigration. *Windrush Betrayal* bears witness to the unfair detention of UK residents, inadequate Home Office documentation procedures, and a failure to correct a series of longstanding procedures that have led to many lawful UK residents losing access to jobs, healthcare, housing and even their country of citizenship. The film not only highlights restricted social and physical mobilities as a central theme, but also negotiates socio-spatial restrictions borne out of pandemic conditions—the film was recorded using multiple cameras by actors in their own homes. It also extends information highlighted in print and online news by illustrating the emotional impacts of mobility constraints and lengthy waiting times for people going through residency and citizenship confirmation processes in the UK. Produced in 2020 during the initial period of lockdown as Covid-19 infections were rapidly increasing, the film incorporates 50 actors who take turns recounting the words of individuals forced to navigate through an increasingly controversial government department and its related procedures, after having been told they were at risk of deportation from the UK. Those targeted were advised to seek legal advice and threatened with potential deportation unless adequate records could be confirmed. Many of those facing possible detention and removal had travelled to the UK as part of the Windrush Generation. Released at a time when most mainstream media were focused on Covid-19, the film’s producers utilised online and terrestrial television broadcasts to draw public attention back to a just-released and highly critical independent report investigating the Home Office, the *Windrush Lessons Learned Review* (March 2020). This latter report had largely been overlooked by media responding to the rapidly changing circumstances in relation to the emerging pandemic.

Actors Lana Joffrey and Martina Laird recruited 49 additional actors (including Lewis Hart, who edited the film), to memorise and record the words of Windrush victims. Each actor directly addresses the camera/viewer with a short statement declaring the name of the person and their age, when they arrived in the UK (often many decades before as children), the varied contributions people had made to the UK (for example working in the National Health Service), and how the Home Office actions had impacted them (loss of jobs, fear, homelessness, separation from family, death). Due to the pandemic many of the actors were available to record at short notice, and the style of recording mirrors the changing aesthetic noted above in relation to changing contexts for television news recordings, where minimalist living rooms become the central story space (each actor recorded their segment separately at home). The film ends with the two statements—the first by the final actor, to the camera: “This has been a great injustice, which will be written about in the history books.” Then followed by a concluding text statement:

Following the *Windrush Lessons Learned* investigation into Home Office practices, Wendy Williams has expressed “serious concerns” that failings in the department “are consistent with some elements of the definition of institutional racism.” Calls for Home Office policy and practice to be scrutinised have so far gone unheeded. As of the end of May 2020, despite Home Secretary Priti Patel repeating an apology made two years earlier by then Home Office Secretary Amber Rudd, only 60 of the 1000s of cases have received any of the compensation to which they are legally and morally entitled (Corradi, 2020).

*Windrush Betrayal* was inspired partly by the *Windrush Lessons Learned* *Review* and by an article written by *Guardian* journalist, Amelia Gentleman (2020a) (and Gentleman, 2019), examining the unfair treatment of British citizens by the Home Office. In her article discussing how “The fury pours from the screen,” Gentleman (2020b), begins her response to the film stating:

Normally, newspaper articles have a very short shelf life—quickly read on the day of publication and subsequently either entirely forgotten or left to linger vaguely in the readers’ subconscious. It is rare for news reports to inspire an artistic response, and almost certainly unprecedented for a piece to result in 50 actors reading out 50 paragraphs from a *Guardian* article.

*The Guardian* as the main dialogue provided by the film’s actors. These statements are combined with research by *The Guardian* documenting the impacts of UK Home Office migration and deportation policies, particularly in relation to the Caribbean Diaspora. The film is available (for free) via *The Guardian’s* website, and is available as a series of five chapters, and a complete film, on the production company, *Valiant Truth*’s (2020) *YouTube* channel. The mobility of the film across different platforms is, therefore, in marked contrast to the restricted mobility of the films’ subjects, making the accessibility and movement of these stories even more pertinent.

Despite the mobility of news stories across different media platforms, when the independent *Windrush Lessons Learned Review* was published, there was limited public engagement with the report. Yet it found comprehensive and fundamental failures in the Home Office's handling of residency and citizenship status confirmations (William’s 2020, 7):

Members of the Windrush generation and their children have been poorly served by this country. They had every right to be here and should never have been caught in the immigration net. The many stories of injustice and hardship are heart-breaking, with jobs lost, lives uprooted and untold damage done to so many individuals and families.

*Windrush Betrayal* bring the harsh reality of these findings to light: spoken directly to camera, engaging with the viewer, we hear the ways in which the Home Office procedures became “like a death sentence” (Joffrey et al., 2020). The film begins with an audio of overlapping voices (all excerpts from the Windrush victim statements)—almost a desperate attempt to get the stories heard—but these are then filtered out, so that in the main body of the film we hear each succinct testimony, one-by-one. The film successfully provides the opportunity to slow down while efficiently critiquing the generalised identities depicted in brief news reports. The film provides a step towards holding government practices to account by illustrating the failures in accountability shown through contradictory government procedures: demanding documentation that was not legally required or had been previously destroyed by the same government agency; declaring the need for people to limit their movements during pandemic restrictions while deporting people from the UK with limited social/legal support (Taylor, 2021); and using citizenship as a punitive identity rather than a basis for government protection. By including numerous uninterrupted stories of individuals facing Home Office scrutiny, the film communicates that these are not exceptional events, but rather that the outrage lies in the way that these have become part of “everyday” traumatic geographies of a governance scandal.

Investigative journalism and documentary film can challenge dominant images of migration through narratives that humanise people facing the threat of deportation and the concomitant impacts on friends and families. In a context in which paper documents are often inaccessible or used as a form of control, film provides an invaluable way to document and give voice to people who have been denied the opportunity to have their story told and heard. In addition, by situating the film in the context of related news sites and being included in digital film festivals and social media platforms, it can reach a wider audience in which additional information can be speedily accessed and interactive discussions held.

Flexible mobilities—both temporally and spatially—are viewed as necessities in the timeliness and in situ character of news coverage. *Windrush Betrayal* connects with this sense of urgency and place, whilst also highlighting the opposite responses in relation to the (lack of) government reform around immigration monitoring. News coverage since the film was released has pointed out that although some limited progress has been made in the processing of compensation claims for citizens and residents who were wrongly told they could be deported, many claims are still outstanding, and several claimants have died while waiting (Gentleman, 2021). Indeed, a more recent cross-party report from the UK Parliament goes even further in its criticism, stating that control for the compensation scheme should be moved from the Home Office, particularly given that “as of the end of September [2021], only 20.1% of the initially estimated 15,000 eligible claimants have applied to the scheme and only 5.8% have received any compensation” (Home Affairs Committee, 2021). This *Windrush Compensation Scheme Report* reignited outrage at the lack of government action and justice, a response which stands in contrast to an apparently emotionally distant Home Secretary and Home Office.

Documentary film deploys and reflects emotion: it presents emotional geographies that provide evocative ways for us to negotiate specific events, testimonies, and places. *Windrush Betrayal* is part of ongoing dialogues that document and challenge the emotional and temporal constraints of news features while offering narrative resistance. The documentary and related media coverage, provide an important contribution towards discussions addressing mobility, borders and the need to personalise media representations of immigration policy, and the relevance of film for more inclusive news coverage.

## Conclusion: Film, media convergence and mobile geographies

“I know how to tell stories, but how do I begin to tell silence” (Miller, 2021, 46).

There are gaps—and silences—in the stories we share and the media we produce and watch. Some of these silences signal unintended exclusions, wilful omissions or experiences that are acutely difficult to articulate. These stories communicate geographies of hope, fear, family and loss. They depict towns where we went to school and made friends, ships where we travelled to a new place of work, living rooms that were places of comfort and family--at times transformed into places of anxiety and uncertainty. These films and spaces are not disconnected: they exist not only in local material spaces, but are interconnected with the practices of organisations, national discourses, structural inequalities, and immigration policies.

Film is diverse and offers an emotive way to engage, anger, cheer and challenge viewers and media producers. This study illustrates the varied ways that film intersects with other media formats and diverse discussions addressing current events, particularly around immigration and changing news contexts. As media formats have increasingly converged, particularly in light of Covid-19 restrictions on approved places for recording and performing, film has provided an integral component of extended coverage of how people experience place and government policies. Changing circumstances—including the impacts of social distancing regulations—and increasingly mobile technologies, mean that the home has become a more visible space in which film content has provided the opportunity to question government depictions of migration and contradictory constraints on mobility.

*Windrush Betrayal* provides an example of a film challenging borders between news studios and the home, and between representation and action. The film’s production is an act of activism that challenges exclusionary UK government depictions of immigration and highlights the importance of individual, yet shared experiences. This film, and other activist media highlight the need for a broader range of voices to be included in immigration policies and mainstream media discussions of mobility and citizenship. Film, in combination with other media formats, provides the opportunity for greater awareness of policy impacts that might otherwise receive limited wider attention. The critical issue is to what extent and in what ways future films can address social injustice and help push towards reparative governance.
